# Selections

**Published:** 1897-09

**Authors:** 


### SELECTIONS.

Referring to poisoning by larkspur or monkshood, Veterinarian Knowles, of Montana, issues the following pertinent suggestions: The most widely distributed species grows in almost every part of the State. This species is without hairs at the base, but with spreading hairs above. The leaves are five-parted, with divisions two or three cleft. The flowers are large, deep blue; the upper petals veined with purple, the spur long and slender. From the observation of several cases of larkspur-poisoning, which happened in Montana early in the spring, at a time when the blossoms and seeds had not yet been produced by the plants, without any doubt the poisonous principle in larkspur is distributed throughout the whole plant (root, leaves, flowers, and seeds), and, therefore, poisoning may occur at almost any time during the life-period of the plant. I ascertained upon close investigation that cattle and sheep are most likely to eat the plant and become poisoned when they are on a range short of grass or when turned immediately into a locality while hungry and in a condition to eat any plant-life in sight.

The common symptoms of poisoning in sheep and cattle are manifested first by the animal straying behind the herd, appearing dull and indifferent to its surroundings, but if suddenly startled will walk in a directly straight line until meeting some obstruction, when it probably falls, makes but a few struggles, lying remarkably quiet under the influence of the poison. There is rarely any bloating or hoven, but in nearly all cases a dribbling of saliva from the mouth, champing of the jaws, and frequent attempts at swallowing.

The treatment most successfully applied has been pouring water of ammonia onto a rag or sponge and holding the same to the animal's nose until it fully inhales the fumes of the ammonia; it sometimes is necessary to pour five or six drops of ammonia into the nostrils. The administration every ten or fifteen minutes to sheep of a teaspoonful of ammonia-water, and the administration of alcohol (in tablespoonful-doses) diluted with three times this quantity of water every fifteen or twenty minutes, will be found beneficial when ammonia does not promptly relieve the animal. Where it is possible, and the drug is accessible, a hypodermic injection of the sixtieth of a grain of atropia sulphate to sheep and one grain to cattle will bring about a cure or relieve the poisoning rapidly, often reviving them when they are apparently beyond help. Digitalis and nux vomica in small doses are also useful, and frequently bring about a cure very promptly.

The *New England Farmer* very effectively answers a correspondent who says "where the eye fails to detect tuberculosis the disease is so infinitesimal as to preclude its danger to consumers of meat and milk."

The disease may advance considerably, even so much as to make it possible that the milk and meat may be diseased before the eye can detect any trouble. Still we admit that tuberculin will also detect the disease in such early stages that the milk and meat at the time of detection are perfectly wholesome; but we must remember that while the animal might grow no worse and might even recover, the chances are that in the majority of cases the disease would increase until both milk and meat might be dangerous, and the animal a loss to its owner. When the danger-point comes and is passed no one can tell.

We do not see how anyone can dispute these fundamental facts. The best method of procedure to be adopted, based upon these facts, is, as the *New England Farmer* has stated many times, a matter of doubt; and unquestionably slightly diseased animals can be isolated and cared for so that they will not contaminate others, and so that they may have many years of safe usefulness; but how much will the average farmer save by going to the expense of isolating such animals and caring for them as would be necessary under the circumstances? Would not the average farmer be about as well off to kill such animals at once, especially if the State will pay part of the expense, as he would be to attempt a course of isolation and doctoring? It is not economy to pay two dollars for the purpose of saving one.

While it may be that the communication of consumption to the human family through the bovine is "extremely improbable," it is a fact that the preponderance of medical sentiment regards the communication as possible to such an extent as to urge many precautions which were not regarded necessary a few years ago.

Vaccination of Tuberculosis.—Richet vaccinated a dog with tuberculous poison which had been taken from a fowl. Some months later he vaccinated this same dog as well as five others with human tuberculous poison. All five latter dogs died of tuberculosis within four months, while the first dog remained continually healthy. A second dog was now handled exactly as the first dog. He, together with five others, was sometime later infected with human tuberculous poison. Again, the five dogs died of tuberculosis in a longer or shorter time, while the first one remained sound. A third time Richet vaccinated another dog, not with fowl-tuberculosis, but with a weak dose of human tuberculous poison. Later this dog, together with another, was infected with large doses of human tuberculous poison. The former remained healthy; the latter died of tuberculosis. That it is not a question here of accidental immunity is proved by the death of the five dogs in the first two experiments. It can only be assumed that immunity is brought about by the weak tuberculous substance. However, this is, alas! not free from dangers; although it is undoubtedly effective, it kills about 50 to 75 per cent. of the animals experimented upon. For the present it cannot, therefore, be utilized in therapeutics.—*Berl. th. Wosch. in Thierarzt. Centralblatt*, November 1, 1896.

